# Antibacterial Activity and Shear Bond Strength of Orthodontic Adhesive Containing Various Sizes of Chitosan Nanoparticles: An In Vitro Study

**DOI:** 10.7759/cureus.54098

**Published:** 2024-02-12

**Authors:** Reem Almeshal, Sarah Pagni, Ala Ali, Driss Zoukhri

**Affiliations:** 1 College of Dentistry, Tufts University, Boston, USA; 2 Department of Public Health and Community Service, Tufts University School of Dental Medicine, Boston, USA; 3 Department of Prosthodontics and Operative Dentistry, Tufts University School of Dental Medicine, Boston, USA; 4 Department of Comprehensive Care, Tufts University School of Dental Medicine, Boston, USA

**Keywords:** white spot lesions, orthodontic adhesive, shear bond strength, streptococcus mutans, chitosan

## Abstract

Introduction: White spot lesions are common after orthodontic treatment. Chitosan nanoparticles (CS-NPs) have emerged as promising antibacterial agents that inhibit the growth of *Streptococcus mutans*. The aim of the study was to investigate the nano-effect of adhesives containing CS-NPs on *S. mutans* and their effects on shear bond strength.

Materials and methods: The inhibitory effects of two sizes of CS-NPs were assessed using the disc agar diffusion method. Four wells were created in the petri dishes, and each was inoculated with broth (negative control), chlorhexidine (positive control), CS-NPs (20 nm), or CS-NPs (131 nm). An Instron machine was used to evaluate shear bond strength by allocating 24 teeth into three groups, and all measurements were recorded in megapascals. Caries progression was assessed using the International Caries Detection and Assessment System and surface profilometry. Statistical analysis was performed using IBM SPSS Statistics for Windows, Version 27.0 (Released 2020; IBM Corp., Armonk, New York, United States) for a one-way ANOVA followed by Tukey’s multiple comparison test.

Results: Disc agar diffusion showed a reduction in *S. mutans* in the CS-NP group compared to the control (p < 0.001), with no statistical significance between the sizes of 20 and 131 nm (p = 0.95). Regarding shear bond strength, no differences were recorded when adhesive-containing CS-NPs and the control were compared (p = 0.44). Additionally, no differences were found within the CS-NP groups (p = 0.91). Caries assessments showed excellent agreement, as indicated by a weighted kappa. Profilometry readings showed higher surface roughness in the control than in the CS-NP groups (p < 0.001), with no statistically significant difference between the CS-NP groups (p = 0.72).

Conclusion: CS-NPs of both sizes tested had similar antibacterial effects. In addition, the incorporation of CS-NPs did not affect shear bond strength.

## Introduction

White spot lesions (WSLs) are characterized by white opacities and are commonly observed in the initial stages of enamel decalcification. Following fixed orthodontic treatment, WSLs can significantly affect patient satisfaction and perception of overall treatment effectiveness [1]. Surface decalcification frequently occurs when the biofilm remains undisturbed for approximately four weeks, primarily due to bacterial accumulation on the tooth surface [2]. The prolonged presence of acidogenic microbes, such as *Streptococcus mutans*, lowers the pH and consequently dissolves hydroxyapatite crystals in the enamel, resulting in the formation of white lesions.

In a study performed by Gorelick et al. [3], researchers showed that among 50 individuals, 27% developed WSLs, as orthodontic appliances such as brackets prevented the maintenance of proper oral hygiene by promoting an optimal environment for acid-producing bacteria. Moreover, a review by Sundararaj et al. [4] indicated that 68% of orthodontic patients exhibit WSLs during treatment. Several factors can predispose patients to WSL development, including poor oral hygiene [5] and the presence of orthodontic appliances [6]. In addition, orthodontic adhesives play an important role in WSL incidence [7]. Factors such as excessive composite, the creation of micro-gaps, and poor bonding techniques lead to adhesive failure and subsequent increased decalcification of the tooth. Sukontapatipark et al. [7] supported these findings using scanning electron microscopy (SEM) imaging of an excessive orthodontic adhesive one week after bracket placement, which revealed a monolayer of bacteria within the extracellular matrix.

In recent years, nanoparticles have integrated and reformed the field of dentistry, offering promising clinical outcomes. The nano-effect of the particles is theorized to possess a greater antibacterial effect on oral biofilm. Nanoparticles such as silver oxide have emerged as a novel strategy for enhancing the clinical outcomes of various dental applications [8]. However, recent studies aimed at identifying more natural sources of nanoparticles have focused on the use of chitin, an organic substance with significant antibacterial properties found in the exoskeletons of crustaceans, fungal cell walls, and insects. The insolubility of chitin limits its potential applicability in the biomedical field. The deacetylation of chitin, discovered by Rouget in 1859 [9], produces a soluble, modified chitin material with the same antibacterial potential as chitin.

Chitosan nanoparticles (CS-NPs) are applicable in a variety of fields and have attracted the attention of many researchers. In the field of dentistry, several studies have reported favorable results when utilizing CS-NPs for various dental applications, including in toothpastes, mouthwash, and root canal sealers, as a drug delivery system [10,11]. Our understanding of the mechanism underlying the effects of CS-NPs remains incomplete. Some have suggested an electrostatic interaction theory, in which positively charged amino groups in nanoparticles interact with negatively charged bacteria, resulting in cell wall disruption, internal component leakage, and subsequent bacterial death [12]. Despite several studies demonstrating the antibacterial effect of CS-NPs, there is a limited understanding of their nano-effect.

Furthermore, it is vital to modify the mechanical properties of orthodontic adhesives when incorporating new materials. Shear bond strength (SBS) is measured as the maximum resistance between the orthodontic bracket and tooth surface before a vertical or horizontal fracture [13]. Ideally, the SBS of an orthodontic adhesive should be great enough to tolerate constant occlusal loading during mastication and forces exerted by orthodontic wires. According to Reynolds [13], orthodontic adhesives should have a SBS within the range of 60-80 kg/cm^2^ (5.9-7.9 MPa) to be considered acceptable in clinical settings. Failure to achieve optimal adhesive strength can compromise treatment outcomes and result in bracket debonding, treatment delays, and enamel damage. Several factors affect the SBS of orthodontic adhesives, such as the type of dental adhesive used, exposure time, and the intensity of the light curing process [14].

This study aims to assess the antimicrobial effect and SBS of an orthodontic adhesive containing CS-NPs of varying sizes. The study hypothesis suggests that smaller nanoparticles will have greater antibacterial activity than larger nanoparticles without affecting the SBS.

## Materials and methods

Preparation of CS-NPs

CS-NPs were prepared using the ionic gelation method described by Calvo et al. [15]. Acetic acid (Fisher Scientific Inc., Waltham, MA, USA) was obtained and used as the solvent for medium molecular-weight chitosan. Sodium triphosphate (TPP, technical grade 85%; Sigma Aldrich, St. Louis, MO, USA) was dissolved in distilled water to produce the chitosan/TPP solution. The solution was then magnetically stirred for an hour to create CS-NPs, which were then centrifuged for four minutes prior to discarding the supernatants. Sonication was performed for approximately three minutes to prevent nanoparticle aggregation.

Characterization of CS-NPs

**Figure 1:**
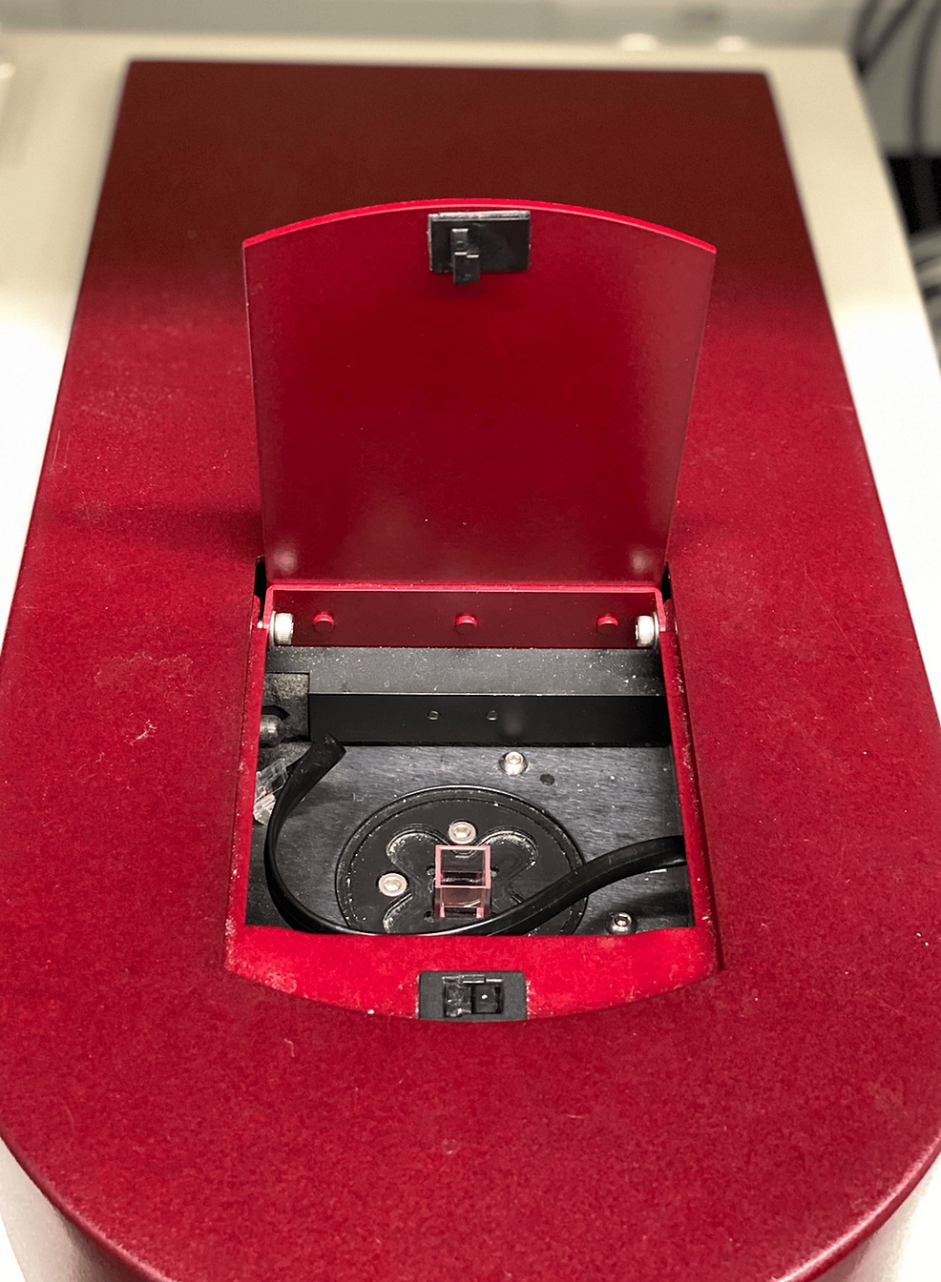
DLS machine used to determine the average particle size of CS-NPs. DLS: dynamic light scattering; CS-NPs: chitosan nanoparticles.

Dynamic light scattering (DLS; ZetaPALS Zeta Analyzer, Brookhaven Instruments Corp., Holtsville, NY, USA) was used to confirm the size distribution of the CS-NPs (Figure 1) [16]. DLS analysis measures fluctuations in the scattered light intensity caused by the motion of particles, providing information about their size. All measurements were performed at the Tufts University Science and Technology Center (Medford, MA, USA).

Bacterial strain growth conditions and collection

The bacterial strain *S. mutans* (ATCC© 25175™) was purchased from the American Type Culture Collection (ATCC, Manassas, VA, USA). The quadrant streak method was used to isolate individual colonies from the culture by streaking the agar plate in the first quadrant using an inoculation loop and then rotating the plate by 90°; this process was repeated until four quadrants were streaked. The plates were aerobically incubated overnight at 37°C. After 24 hours of incubation, bacterial growth was confirmed by visual inspection of the agar plates. A colony was then collected using a loop, added to 5 mL of brain-heart infusion (BHI) broth, and subsequently incubated for 24 hours at 37°C for further use.

Sample preparation

To ensure sufficient statistical power, G*Power Ver (3.1) indicated that with an effect size of 0.80 and an alpha level of 0.05 would provide 90% statistical significance for a sample size of 24 teeth. The research was approved by the Dental Research Administration (Registration number 2020-B75) at Tufts University School of Dental Medicine. Consent forms were waived due to the use of unidentified data and the nature of this study. Twenty-four extracted lower central incisors were obtained from the Department of Oral and Maxillofacial Surgery. Lower incisors were selected for this study because of their availability at the time of sample collection. Teeth with any enamel alteration, such as cracks or previous restorations, were excluded from the study. After that, the teeth were immersed in a 10% formalin solution for five days. The roots were then resected using a precision saw (Isomet 1000; Buehler, Lake Bluff, IL, USA). Additionally, the enamel surface was treated using the etch-and-rinse technique. The enamel was etched by applying 35% phosphoric acid (Ultra-Etch, Ultradent Products Inc., South Jordan, UT, USA) for 15 seconds and then carefully rinsed for 10 seconds. Next, a primer (3M Unitek Transbond XT, Monrovia, CA, USA) was applied to the enamel surface of the teeth and polymerized at 1100 mW/cm^2 ^for 30 seconds with an LED light curing device (Demi Plus; Kerr Dental, Orange, CA, USA).

Subsequently, orthodontic adhesive containing CS-NPs was prepared using the method described by Mirhashemi et al. [17], and a blend of 300 mg of the adhesive (3M Unitek Transbond XT, Monrovia, CA, USA) was incorporated with CH-NPs (7.5 mg) to achieve a concentration of 2.5%. The formulation was placed on a glass slab using a mixing spatula until a uniform thickness was achieved, and all processes were performed in a semi-dark room. Thereafter, extracted teeth were randomly divided into three groups: Group A (control [regular adhesive]), Group B (adhesive with 20 nm CS-NPs), and Group C (adhesive with 131 nm CS-NPs). The sizes of nanoparticles tested were selected to address a gap in current research and test the nano-effects of CS-NPs at opposite ends of the size spectrum. Adhesives were then applied to enamel surfaces with uniform orthodontic metal brackets (Forestadent, St. Louis, MO, USA) and light-cured for 30 seconds to ensure a standardized distance of 2 mm from the light-cure tip to the bonding surface.

Preparation of a saliva-like buffered solution

To develop a biomimetic model relevant to clinical conditions, a saliva-like buffer was prepared using a mixture of 0.147 g/L calcium chloride (CaCl_2_· 2H_2_O), 0.041 g/L magnesium chloride (MgCl_2_ · 6H_2_O), 0.025 g/L sodium bicarbonate (NaHCO_3_), 0.544 g/L potassium phosphate (KH_2_PO_4_), and 2.237 g/L potassium chloride (KCl) dissolved in distilled water. The solution was then thoroughly mixed using a magnetic stirrer for 10 minutes, autoclaved for sterilization, and stored at 4°C.

Disc agar diffusion test

To evaluate the antibacterial effectiveness, disc agar diffusion was utilized by creating three BHI plates. A tube containing *S. mutans* culture was diluted as previously described. Thereafter, 100 µL of the diluted bacterial culture was added and spread uniformly across the surface of each plate. Plates were then incubated at 37°C for 30 minutes. To ensure uniformity, four wells were created in the agar material using a cork borer, and 80 µL of the following was added to wells: Group A (negative control), BHI broth; Group B (positive control), 0.12% chlorhexidine (CHX) [18]; Group C, 20 nm CS-NPs; and Group D, 131 nm CS-NPs. Plates were incubated for 24 hours at 37°C. Thereafter, zones of inhibition were measured using a ruler to evaluate antibacterial effects. The experiment was repeated in triplicate to ensure the reliability and validity of the results.

SBS assessment

**Figure 2:**
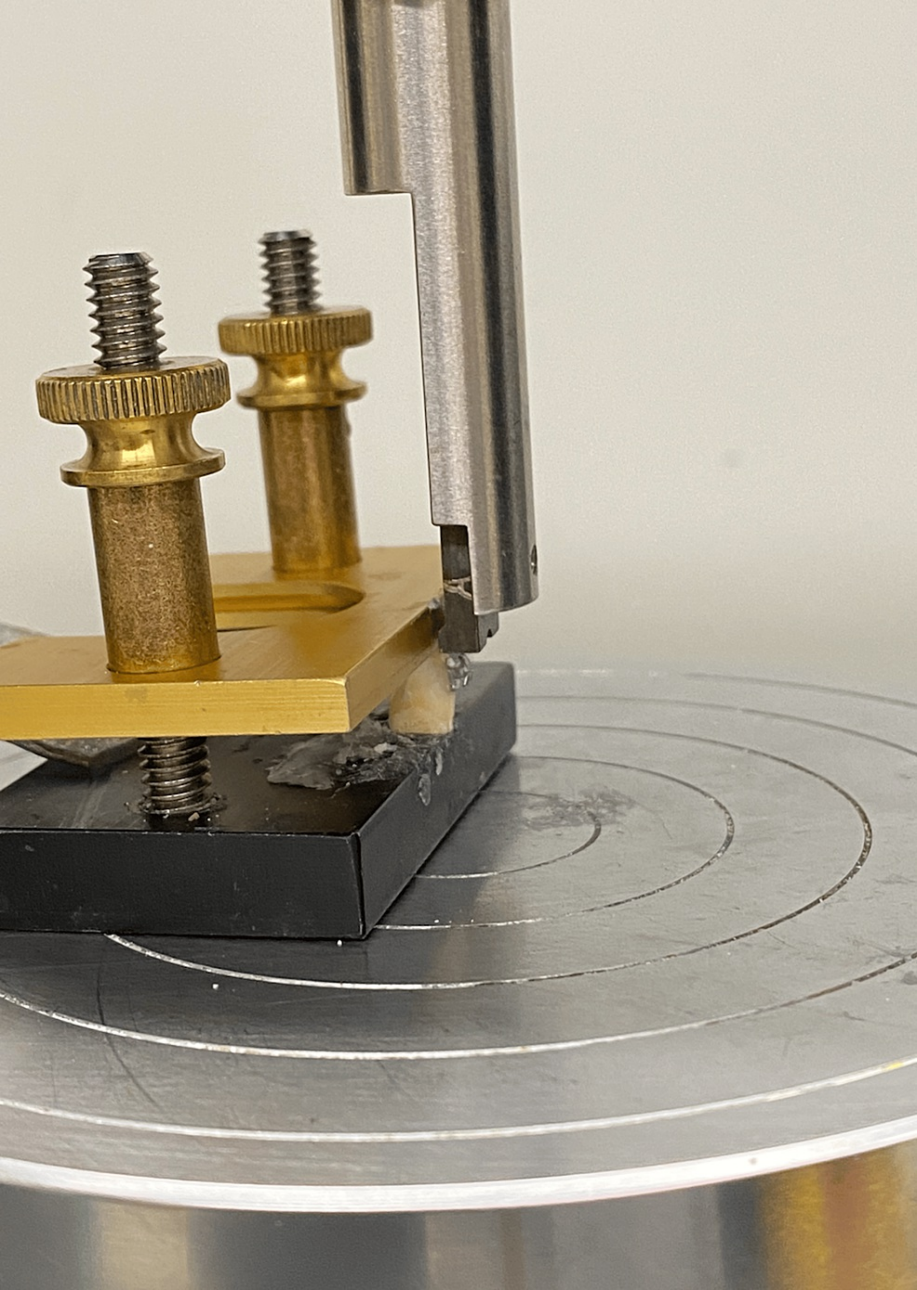
SBS test using Instron Universal Machine, with debonding tip. SBS: shear bond strength.

Extracted teeth with bonded brackets were carefully placed in a 24-well plate containing a mixture of 100 µL of the adjusted *Streptococcus* suspension and 1,000 µL buffered saliva. The plate was then incubated at 37°C, with media exchanged every 48 hours throughout a five-week period. After five weeks, the biofilm was removed by wiping the teeth with sterilized gauze and cleansed with a water syringe. SBS was evaluated using a universal testing machine (Instron 5566; Instron Corp., Norwood, MA, USA). Teeth were positioned to ensure that the force was parallel to the gingival-occlusal direction (Figure 2). The controlled crosshead speed was set to 1 mm/min, and a 1 kN load cell was used. A gradual force was applied until the bracket separated from the enamel. At this point, compression stress at break was recorded and expressed in megapascals (MPa).

Caries progression assessment

Following the removal of the brackets, the teeth were divided into three groups of eight. WSLs were visually assessed under a stereomicroscope (SZX16, Olympus) at 4× magnification. Two calibrated investigators, blinded to each other’s assessments, utilized the International Caries Detection and Assessment System (ICDAS) to categorize observed demineralization under the same conditions. The following codes were used to classify teeth based on demineralization levels: Code 0 (sound tooth surface), Code 1 (first visual change in enamel, such as an opaque white spot), Code 2 (distinct visual change in enamel, with no cavity or break in the surface), Code 3 (localized enamel breakdown), Code 4 (dentin involvement), Code 5 (visible dentin involvement), and Code 6 (extensive cavity) [19]. Interrater agreement was analyzed using the weighted Cohen’s kappa.

Evaluation of surface roughness

Following the visual examination of the teeth, surface profilometry (DekTak XT Profilometer) was used to examine enamel roughness [20]. Profilometer parameters were set as follows: 3 mg of force, 2 µm stylus radius, 20 seconds scanning time, and a resolution of 0.111 µm. Three measurements were taken at the center of each sample, with the average roughness value calculated (µm).

Statistical analyses

Data obtained were analyzed using IBM SPSS Statistics for Windows, Version 27.0 (Released 2020; IBM Corp., Armonk, New York, United States). Descriptive statistics, including means and standard deviations, were calculated. Normality was confirmed using QQ plots, and the homogeneity of variance was assessed using Levene's test, with a level of significance of 5%. A one-way analysis of variance (ANOVA) was performed to compare the mean zone of inhibition diameter, SBS, and surface roughness values of the groups. Tukey’s was used as the post hoc test to differentiate the significant difference. Interrater agreement of ICDAS scores was assessed using the weighted kappa statistic.

## Results

Characterization of CS-NPs

**Figure 3:**
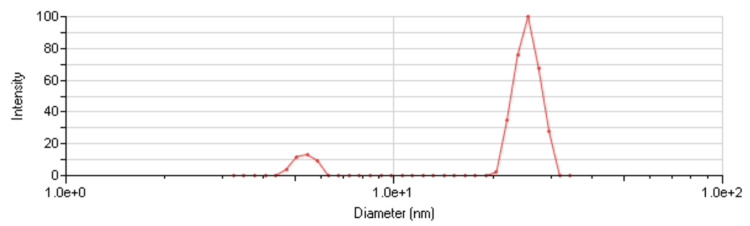
Size distribution of the smaller particles after suspension in distilled water. The peak represents a mean particle size of 20 nm.

**Figure 4:**
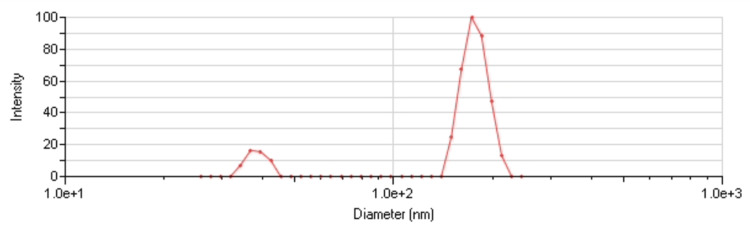
Size distribution of the larger particles after suspension in distilled water. The peak represents the mean particle size of 131 nm.

The size distribution of the nanoparticles in each sample was assessed using DLS analysis, revealing mean particle sizes of 20 and 131 nm (Figures 3, 4).

Disc agar diffusion test

The disc agar diffusion test revealed a statistically significant difference between the control and CS-NP groups (p < 0.001), indicating that CS-NPs had an inhibitory effect on *S. mutans*. Moreover, a comparison of both CS-NP groups revealed that smaller CS-NPs (20 nm) had a slightly greater zone of inhibition than larger CS-NPs (131 nm); however, this difference was not statistically significant, p = 0.95 (Tables 1, 2). Additionally, among all materials tested, CHX had the greatest antibacterial effect on *S. mutans*.

**Table 1:** Descriptive statistics for the disc agar diffusion test, expressed as the mean and SD in mm. CHX: chlorhexidine; CS-NPs: chitosan nanoparticles; SD: standard deviation.

Group	Mean ± SD (mm)	Median
CHX	32.44 ± 7.49	30.66
20 nm CS-NPs	19.55 ± 2.50	19.66
131 nm CS-NPs	19.44 ± 2.52	18.33

**Table 2:** Multiple comparison of antibacterial effects of CS-NPs on S. mutans disc agar diffusion. CHX: chlorhexidine; CS-NPs: chitosan nanoparticles. *Statistical significance <0.05. ***Statistical significance <0.001.

Group		p value
Control	CHX	<0.001***
Control	20 nm CS-NPs	<0.001***
Control	131 nm CS-NPs	<0.001***
CHX	20 nm CS-NPs	0.047*
CHX	131 nm CS-NPs	0.046*
20 nm CS-NPs	131 nm CS-NPs	0.958

Shear bond strength

The mean SBS of the control group was 14.26 MPa and ranged from 10.11 to 19.24 MPa. For the CS-NPs (20 nm), the mean SBS value was 13.13 MPa and ranged from 10.89 to 17.01 MPa. For CS-NPs (131 nm), the mean SBS value was 12.5 MPa and ranged from 8.09 to 17.90 MPa (Table 3). No statistical difference was observed when the mean SBS values of the control adhesive and those of treatment groups containing CS-NPs were compared (p = 0.44; Table 4). In addition, no statistically significant difference was observed when the CS-NP groups were compared (p = 0.91; Table 4).

**Table 3:** Descriptive statistics for the SBS, expressed as the mean and SD in MPa. CS-NPs, chitosan nanoparticles; SBS: shear bond strength; SD, standard deviation.

Group	N	Mean ± SD (MPa)	Median
Control (no treatment)	8	14.26 ± 2.93	14.53
20 nm CS-NPs	8	13.13 ± 1.91	12.92
131 nm CS-NPs	8	13.3 ± 2.69	11.57

**Table 4:** Intergroup multiple comparison for SBS. CS-NPs: chitosan nanoparticles; SBS: shear bond strength.

Group		p value
Control (no treatment)	20 nm CS-NPs	0.961
Control (no treatment)	131 nm CS-NPs	0.447
20 nm CS-NPs	131 nm CS-NPs	0.913

Caries progression assessment

Overall, findings revealed a lower incidence of WSL in the treatment group than in the control group. A score of 0, representing a sound tooth, was more common among 20 nm CS-NPs (62.5%). Additionally, a score of 1, demonstrating a visual alteration in the enamel, was also observed in teeth provided with 20 nm CS-NPs (37.5%). The control group showed the highest degree of damage, with a score of 2, indicating distinctive enamel changes observed in 62.5% of teeth. The weighted kappa value was 0.875, indicating excellent interrater agreement between the two evaluators according to Fleiss’s interpretation.

Surface profilometry findings

The surface roughness values of both CS-NP groups were significantly lower than those of the control (p < 0.001). There was no statistically significant difference observed when both CS-NP groups were compared (p = 0.72; Tables 5, 6).

**Table 5:** Descriptive statistics for the surface roughness, expressed as the mean and SD in µm. CS-NPs: chitosan nanoparticles; SD: standard deviation.

Groups	N	Mean ± SD (µm)	Median
Control (no treatment)	8	62.62 ± 8.45	60.745
20 nm CS-NPs	8	43.78 ± 9.51	47.33
131 nm CS-NPs	8	47.32 ± 9.23	46.75

**Table 6:** Enamel surface roughness comparison of the control group and CS-NP groups. CS-NPs: chitosan nanoparticles. **Statistical significance <0.01. ***Statistical significance <0.001.

Group		p value
Control (no treatment)	CS-NPs 20 nm	<0.001***
Control (no treatment)	CS-NPs 131 nm	0.008**
CS-NPs 20 nm	CS-NPs 131 nm	0.720

## Discussion

Several studies have investigated the use of a combination of orthodontic adhesives and nanoparticles to reduce WSLs [21]. Notably, CS-NPs have showed promise due to their significant inhibition of the growth of *S. mutans*, a bacterium commonly associated with dental caries. Nanoparticles are defined as particles ranging from 1 to 100 nm in size. Several factors affect particle size, such as chitosan concentration, chitosan-TPP mass ratio, and molecular weight [22]. In this study, by modifying the CS/TPP ratio, we obtained particle sizes of 20 and 131 nm. Size selection was formulated to address the research gap and test the nano-effect of CS-NPs. The nanoparticle sizes used in this study were similar to those reported by Martínez-Robles et al. [23], who observed a size-dependency association of the AgNPs and their antibacterial efficacy against *S. mutans*.

To assess the antibacterial activities of the particles, a DAD test was used to confirm the inhibitory effect of CS-NPs on *S. mutans* growth. Although increased bacterial growth inhibition was observed after treatment with the smallest nanoparticles (20 nm), this difference was not statistically significant. These findings align with those of a study by Zhu et al. [24] who observed a significant reduction in growth curve when CS-NPs ranging from 1.81 to 18.94 nm and 6.47 to 48.72 nm were used on *Escherichia coli* as well as *Staphylococcus aureus*. Additionally, a study conducted by Mirhosseini et al. [25] utilized similar nanoparticle sizes (20 nm, 40 nm, 140 nm) of zinc oxide on four different oral microorganisms: *S. mutans*, *Enterococcus faecalis*, *Lactobacillus fermentum*, and *Candida albicans*. The largest zone of inhibition was recorded with the smallest zinc oxide nanoparticles (20 nm) in comparison with other groups. These studies can indicate that the antibacterial effects of CS-NPs depend on the size of the nanoparticles, as it is theorized that smaller nanoparticles may penetrate the bacterial cell wall more effectively, leading to enhanced cellular death compared to large nanoparticles.

Preservation of enamel integrity is an important consideration when integrating antibacterial agents. After debonding orthodontic brackets, the enamel surface should be free of caries, adhesive remnants, and microcracks. The ICDAS was used to evaluate lesion depth at the visual level at the earliest stage. In the current study, the control group had more WSLs than did the CS-NP group. This finding aligns with those of Zhang et al. [26], who demonstrated that enhanced surface remineralization was observable after using chitosan to pretreat enamel surfaces. The electrostatic theory may explain the latter results as superior antibacterial effects were observed with Gram-positive or Gram-negative bacteria, depending on the interactions between the cell wall components and CS-NPs.

To further assess the caries progression, the surface roughness of the enamel was measured. Assessing the surface roughness of enamel samples provides insight into the progression of caries. Deeper WSLs typically exhibit increased surface roughness and porosity, features that lead to plaque retention and external staining [27]. In this study, our assessment of surface roughness revealed that there were fewer surface irregularities in the CS-NP group than in the control group; however, no significant difference between 20 and 131 nm CS-NPs was observed, findings consistent with those of the DAD test. 

Moreover, incorporating an adhesive into a substance that possesses antibacterial properties requires a careful evaluation of bond strength. Both high and low SBSs negatively affected the treatment outcome. Excessive SBS exceeding 60 MPa may potentially damage the enamel surface upon debonding the brackets. A study by Scribante et al. [28] found that the control group exhibited a significantly higher SBS than the treatment groups; however, all scores fell within an acceptable range. In contrast, a lower SBS can lead to debonding, consequently delaying treatment [29]. Our results showed that no differences in SBS were recorded among the groups tested. Mean SBS values were 10-19 MPa for the control, 10-17 MPa for smaller CS-NPs (20 nm), and 8-17 MPa for larger CS-NPs (131 nm). These results confirmed those reported by Sorourhomayoun et al. [30], who found no significant differences in SBS among five groups of orthodontic adhesives blended with CS-NPs of various concentrations and molecular weights. These results may imply that the incorporation of CS-NPs into orthodontic adhesives has a negligible to insignificant effect on the mechanical properties.

The limitations of this study include not simulating the oral flora and disregarded oral hygiene habits in caries progression. Furthermore, it is important to note that the study was carried out using a single concentration of CS-NPs, which limits the generalizability of the findings. Further studies should compare WSLs associated with a wider range of particle sizes and CS-NP concentrations.

## Conclusions

It can be concluded that (1) the incorporation of CS-NPs in an orthodontic adhesive can significantly inhibit the growth of *S. mutans*, with no differences in microbial activities of both sizes of CS-NPs, and (2) the addition of CS-NPs in the orthodontic adhesive did not adversely affect adhesive SBS. These findings indicate that CS-NPs have potential as a promising adjunct in dental adhesive formulations, improving antimicrobial activity without compromising adhesive properties.
